# A strain-variable bacteriocin in *Bacillus anthracis *and *Bacillus cereus *with repeated Cys-Xaa-Xaa motifs

**DOI:** 10.1186/1745-6150-4-15

**Published:** 2009-04-21

**Authors:** Daniel H Haft

**Affiliations:** 1 The J. Craig Venter Institute, 9704 Medical Center Drive, Rockville, MD 20850, USA

## Abstract

Bacteriocins are peptide antibiotics from ribosomally translated precursors, produced by bacteria often through extensive post-translational modification. Minimal sequence conservation, short gene lengths, and low complexity sequence can hinder bacteriocin identification, even during gene calling, so they are often discovered by proximity to accessory genes encoding maturation, immunity, and export functions. This work reports a new subfamily of putative thiazole-containing heterocyclic bacteriocins. It appears universal in all strains of *Bacillus anthracis *and *B. cereus*, but has gone unrecognized because it is always encoded far from its maturation protein operon. Patterns of insertions and deletions among twenty-four variants suggest a repeating functional unit of Cys-Xaa-Xaa.

This article was reviewed by Andrei Osterman and Lakshminarayan Iyer.

## Findings

Many bacteria produce bacteriocins, toxins built by post-translational modification of ribosomally produced precursors [1]. Recently, Lee, et al. [2] described a widespread type of biosynthesis gene cluster for producing a subclass of bacteriocins with thiazole or oxazole heterocyclic rings. Four hallmarks help identify these systems. First, the toxin precursor itself is generally small, at 50 to 70 residues, such that it may be missed by *ab initio *gene finders. The N-terminal region is a leader peptide that may show homology from one precursor to another. The C-terminal region, however, contains low-complexity sequence, often repetitive and rich in Cys, Ser, or Thr, that may be hard to distinguish from spurious translations of miscalled genes. Second, a putative cyclodehydratase encoded by a neighboring gene joins the side chain of a Cys, Ser, or Thr residue to the carbonyl group contributed by the immediately N-terminally adjacent residue, with the elimination of a water molecule, to form a heterocyclic ring. Third, a dehydrogenase is encoded in the cluster. The dehydrogenase acts on the resulting thiazoline (Cys) or oxazoline (Ser or Thr) ring to produce a thiazole or oxazole ring. Lastly, a protein described as a docking scaffold appears in the same cluster. Additional functionally connected genes may co-occur in these clusters, including putative transporter subunits, immunity proteins, proteases, and enzymes for other types of modifications, but none is required.

We developed hidden Markov model (HMM) search models [3] for the TIGRFAMs database [4] that would identify subsets of the cyclodehydratases (TIGR03603), thiazole/oxazole-producing dehydrogenases (TIGR03605), and scaffolding proteins (TIGR03604), using clusters described by Lee, et al. [2] as the initial standard of truth. These models are built for high specificity, with consideration of local gene context for putative family members used in the manual adjustment of HMM seed alignment membership and scoring cutoffs. Any hit to one of these HMMs has a strong (though difficult to quantify) likelihood of marking a heterocycle-containing bacteriocin biosynthesis-like cluster, while two that are adjacent indicate an even stronger likelihood.

In the genome of *Bacillus anthracis *[5], the causative agent of anthrax, we found a putative bacteriocin-biosynthetic dehydrogenase BA_1266 adjacent to a scaffolding protein BA_1267, but with no candidate toxin precursor ORF nearby. Adjacent to the orphan pair was an uncharacterized protein, BA_1268, with no direct pairwise sequence relationship detected to any cyclodehydratase identified in the work of Lee, et al. However, PSI-BLAST [6] with a fairly stringent cutoff (0.000001) by the third round reached the thiamine biosynthetic enzyme ThiF involved in thiazole formation [7], as well as molybdopterin biosynthesis enzymes and bacteriocin-biosynthetic cyclodehydratases proposed by Lee, et al. This left a three-gene operon, well-conserved in the genus *Bacillus*, with no candidate bacteriocin precursor gene nearby. This observation suggested that the toxin precursor gene might be present in the genome but at some distance from the maturation cluster.

At a distance of 1.27 megabases from the cluster, we identified an 88-residue protein in *B. anthracis *Ames, BA_2677 (GB|AAP26522), with sequence features appropriate for a heterocycle-containing bacteriocin precursor. In particular, the C-terminal region has an exaggeratedly low amino acid complexity. It is Cys-rich, Gly-rich, and highly repetitive; the thirteen Cys residues are regularly interspaced with either two amino acids (ten times) or three amino acids (twice).

Only a fraction of the putative thiazole-containing bacteriocins put forward by Lee, et al. have extended Cys-rich repetitive regions, but such regions are highly suggestive in those that do, such as the Cys-Arg-Gly repeats in a precursor from *Bradyrhizobium japonicum*. We constructed HMMs from alignments of constructed Cys-rich repetitive sequences with periodicities of two (Cys-Xaa) or three (Xaa-Cys-Xaa), repeated four to eight times. The default behavior of the hmmbuild program in HMMER 2.0 is to detect largely redundant sequences and replace actual counts of the different residues seen at each position by "effective counts." Overriding this behavior with the --noeff option forces the use of actual counts rather than effective counts in Cys-only columns of the alignment, sharpening the distinction between Cys itself and all other amino acids. The resulting series of HMMs, based on from four to eight repeats of the tripeptide sequence Xaa-Cys-Xaa, consistently reported BA_2677 as the top or only match in the genome. Searches based on repeated Cys-containing dipeptide repeats, as in the *Staphylococcus aureus *RF122 bacteriocin precursor YP_416854, found no candidate bacteriocin in *B. anthracis*.

The high interest in *B. anthracis *as a spore-forming lethal pathogen with biowarfare capability, and the medical significance of the *B. cereus *group in both infection and food poisoning [8], have helped drive the sequencing of a large number of strains of *B. anthracis*, *B. cereus*, and other *Bacillus *species. Therefore, numerous putative orthologs could be found in completed genomes in the genus *Bacillus*. In each case, a member was found only if and only if orthologs to BA_1266 (dehydrogenase), BA_1267 (scaffolding protein), and BA_1268 (cyclodehydratase) were present in the same genome. In *Bacillus cereus *ATCC 14579, with two such sets of maturation proteins, two putative protoxins were found. More commonly, however, protoxins appear to be accommodated by a single maturation protein cassette, consistent with the broad specificities inferred from heterologous expression studies.

An HMM was built from the N-terminal region of collected BA_2677 homologs in order to represent the presumptive leader sequence. This HMM identified a region of sequence similarity to a previously uncalled putative bacteriocin precursor gene in *Bacillus licheniformis *ATCC 14580. A non-overlapping region of the same open reading frame represents the best match in the genome to HMMs based on Cys-Xaa-Xaa repeats. The respective candidate bacteriocins from *B. anthracis *and *B. licheniformis *are similar in Cys content and spacing, if not in their interleaving sequence residues; triplets of Cys-Gly-Gly in *B. anthracis *are largely replaced by triplets of Cys-Trp-Ser in *B. licheniformis*. Interestingly, the tetrapeptide Cys-Trp-Ser-Cys also occurs in a known bacteriocin, thuricin S [9], within a larger region containing four Cys residues spaced CXXCXXCXXC, although the significance of that local sequence similarity is unclear.

**Figure 1:**
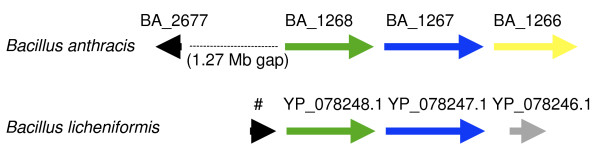
**Genomic context shows evidence for a "heterocycloanthracin" group of bacteriocin precursors**. This shows comparable genomic regions in *Bacillus anthracis *Ames and *Bacillus licheniformis *ATCC 14580. Black signifies proposed protoxin, green for cyclodehydratase, blue for docking scaffold, yellow for dehydrogenase, and gray a *Bacillus*-specific unknown protein. The # character indicates a gene prediction we designate NT03BL1107.1, missed in public annotation.

The *B. licheniformis *gene, however, is adjacent to two appropriate maturation genes, AAU22609 (scaffolding) and AAU22610 (cyclodehydratase). A reciprocal search, initiated from the N-terminal 35 amino acids from *B. licheniformis*, performed by PSI-BLAST (E-value threshold 100 in the first round), finds BA_2677 immediately as the top hit (39% identity, but the E-value is 22 because the sequence is short). PSI-BLAST converges in the third round to recover 24 non-identical sequences closely related to BA_2677 in the leader sequence region, and it finds no other sequences. These results suggest that BA_2677, and TIGRFAMs model TIGR03601 as an HMM for its detection, represent a new family of heterocycle-containing bacteriocins of the class described. The supporting evidence includes bidirectional best hit matches between the leader sequence regions, similar Cys-rich tripeptide repeat structures in the C-terminal regions, and colocalization of the *B. licheniformis *member with two distinctive components, the cyclodehydratase and the scaffolding protein, of canonical heterocycle biosynthesis cassettes. Figure 1 illustrates the comparable genomic contexts.

**Figure 2:**
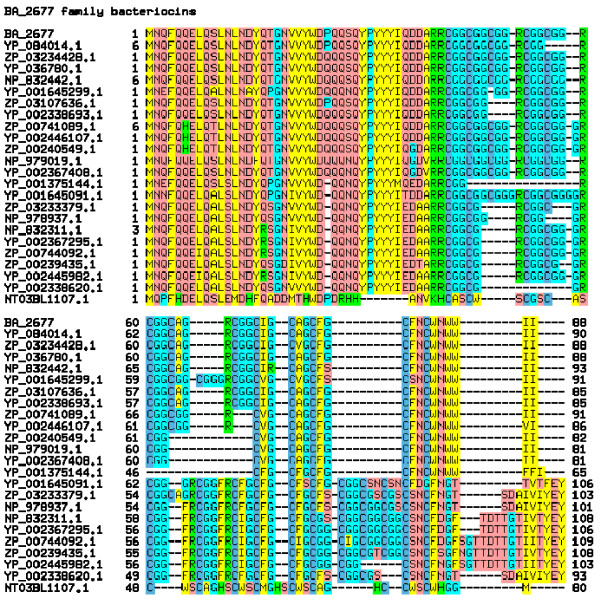
**Sequence alignment shows evidence for a "heterocycloanthracin" group of bacteriocin precursors**. This shows an alignment of protoxin sequences. Insertions and deletions of the form Cys-Xaa-Xaa, and Cys-Gly-Gly in particular, account for many of the differences between non-identical sequence pairs. Shown are *Bacillus anthracis *protein BA_2677 (RF|YP_019318.1), twenty-two non-identical close homologs from other Bacillus strains, and the more distantly related protoxin from *B. licheniformis*. The Arg-Arg pair represents the boundary between the N-terminal (leader peptide) and C-terminal (Cys-rich) regions. At least one heterocycloanthracin is found in every completed genome studied from *B. anthracis*, *B. cereus*, *B. thuringiensis*, and *B. weihenstephanensis*. Strains of origin are *B. anthracis *Ames (sequence 1), *B. cereus *E33l (2), *B. c. *H3081.97 (3), *B. thuringiensis sv. konkukian *str. 97–27 (4), *B. c. *ATCC 14579 (5, 18), *B. weihenstephanensis *KBAB4 (6,15), *B. c. *NVH0597-99 (7), *B. c. *AH187 (8, 23), *B. t. sv. israelensis *ATCC 35646 (9, 20), *B. c. *G9842 (10, 22), *B. c. *G9241 (11, 21), *B. c. *ATCC 10987 (12, 17), B. c. B4264 (13, 19), *B. c. subsp. cytotoxis *NVH 391-98 (14), *B. c. *AH1134 (16), and *B. licheniformis *ATCC 14580 (24).

Figure 2 shows a multiple sequence alignment of the proposed bacteriocin family. The strain-to-strain variability of this putative bacteriocin family presents an opportunity to study patterns of sequence difference for insights into evolutionary pressures and constraints. Examination of sequences in the multiple alignment shows not only that Cys tends to occur every third residue in the carboxyl-terminal region, but also that differences between closely related sequences commonly feature tripeptide indels. We performed counts of all tripeptides among the 23 different sequences from *B. anthracis*, *B. cereus*, *B. thuringiensis*, and *B. weihenstephanensis*. The most abundant tripeptide was CGG (152), outnumbering its phase-shifted alternatives GGC (95) and GCG (76), as well as RCG (83). The multiple alignment therefore is edited [10] in Cys/Gly-rich regions to favor showing Cys-Gly-Gly as the common inserted or deleted tripeptide. Inspection of the alignment shows length variability in the Cys/Gly-rich region between the leader peptide region and two versions of the extreme C-terminal motif, WNWWII and [FY]EY. Despite length differences internally, there are no examples of N-terminally or C-terminally truncated sequences.

The primary function of most bacteriocins is bacteriocidal activity against closely related species or strains. However, these secondary metabolites may have important roles in pathogenesis, in food preservation, as antibiotics, or as pharmacologically active compounds that may lead to the development of new drugs. Since the maturation proteins of heterocyclic bacteriocin have been shown to act rather freely on protoxins from other bacterial lineages [2], it is reasonable to propose that modification can also occur at multiple sites in a single repetitive precursor. The heterogeneity of BA_2677 homologs therefore may correspond to a panel of proteins that, when fully processed, are extensively modified. We suggest the name heterocycloanthracin for this collection of putative bacteriocins related to BA_2677.

## Abbreviations

HMM: hidden Markov model; ORF: open reading frame.

## Competing interests

The author declares no competing interests.

## Authors' contributions

DHH performed the analyses, interpreted the results, and wrote the paper.

## Reviewers' comments

### Reviewer 1

Andrei Osterman, Burnham Institute for Medical Research, La Jolla, CA.

A short article by D. Haft provides us with another beautiful illustration of the power of comparative genomic approach for pathway reconstruction and gene discovery. This study builds on top of a recently published analysis of widespread genomic distribution of pathways generating bacteriocins, short proteins containing oxazole and/or thiazole heterocycles. By combining highly sensitive profile-based search models with the analysis of genome context, D. Haft predicted the existence of a putative bacteriocin maturation pathway in an important group of Bacillus (B. anthracis and B. cereus). The most challenging aspect of this analysis was a "missing" bacteriocin precursor, which in all previously described cases was located in the same chromosomal cluster with genes involved in its posttranslational modification. A convincing prediction of a remotely located gene candidate for a novel version of protoxin characteristic of this group is the most impressive intellectual breakthrough of this elegant study. The identification of an uncalled gene encoding an ortholog of the "heterocycloanthracin" precursor in a chromosomal cluster with the maturation pathway genes in B. licheniformis strongly validated this ambitious inference. A unique and highly attractive feature of D. Haft's article is that it provides biologists with a mature hypothesis that may (and most certainly will) be immediately tested by a focused experiment.

### Reviewer 2

Lakshminarayan Iyer, NCBI

The author describes a bacteriocin gene family from Bacillus species that is not in the vicinity of its predicted maturation genes. As bacteriocins are generally short, can be missed during gene annotation, and not very conserved in sequence, isolating them is of some importance. The main point of this study is that while most bacteriocins are encoded next to the genes involved in their maturation, this particular family, barring one exception, appears to be encoded far away from them. I have confirmed the results independently and agree, based on the evidence from the *B. licheniformis *gene, that these are bacteriocin genes.

I have a few minor comments.

- I recommend adding the source of the sequences in the figure for better clarity. Typically the species names are given as abbreviations.

- The parameters of the PSI-BLAST search using the predicted *licheniformis *bacteriocin as a starting point and the database/s against which it was searched should be mentioned for reproducibility, as a simple web PSI-BLAST search against nr does not yield the result as described in the text. However, upon searching against individual genomes, the bacteriocin sequences are retrievable.

### Author's response

The strain of origin for every sequence has been added to the legend for figures 1 and 2. This clarifies that the twenty-four non-identical sequences represented in figures 1 and 2 include contributions of one each from eight strains, and two each from more eight strains. Just one of the 16 strains shown has two sets of heterocyclic bacteriocin maturation proteins, and it belongs to the group with two protoxins. This observation is consistent with earlier observations that maturation enzymes can show activity on heterologously expressed protoxins. A single maturation cassette may act on two different toxin precursors.

Because of the small size of the putative protoxin, especially once repetitive sequence is removed to leave only 35 N-terminal amino acids, PSI-BLAST requires an E-value more permissive than the default value of 10. The proper search parameters are now described explicitly in the text.
